# Studies on incidence and evaluation of the closed medial patellar desmotomy in lateral recumbency in bovines

**DOI:** 10.14202/vetworld.2015.221-224

**Published:** 2015-02-23

**Authors:** Ajit Kumar Singh, A. K. Gangwar, Kh. Sangeeta Devi, H. N. Singh

**Affiliations:** Department of Veterinary Surgery and Radiology, College of Veterinary Science and Animal Husbandry, Narendra Deva University of Agriculture and Technology, Kumarganj, Faizabad, Uttar Pradesh, India

**Keywords:** bovines, desmotomy, incidence, recumbency

## Abstract

**Aim:**

The present study was conducted to find out the incidence and to evaluate the effectiveness of medial patellar desmotomy (MPD) in lateral recumbency in bovines.

**Material and Methods:**

One hundred and fifteen clinical cases of upward fixation of the patella in cattle and buffaloes were treated by closed MPD in lateral recumbency. Probable etiologies, symptoms and site of surgery including disease occurrence with respect to species, sex and season were also recorded.

**Results:**

In the present study, the highest incidence was reported in bullocks. A high success rate was obtained with the closed method in lateral recumbency.

**Conclusion:**

Based on the findings of this study, it can be concluded that the bullocks were more prone to upward fixation of patella and symptoms were exaggerated in winter season. Closed method of MPD was more suited in both cattle and buffaloes.

## Introduction

**Figure-1:**
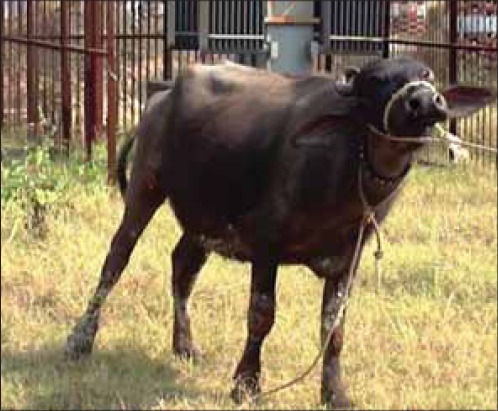
Animal showing typical signs of upward fixation of patella.

Upward fixation of the patella is common surgical problem in Indian cattle and buffaloes [1]. It is characterized by temporary or permanent dislocation of the patella from its regular position with difficulty during locomotion [2]. This condition will not endanger the life of the animal; however it does cause temporary disability and discomfort reducing the economic value of the affected animal [3]. The animals suffering from the problem of upward fixation of the patella of both the legs become a burden to the farmer. Many times treatment is not provided in time due to lack of skilled personnel for surgical treatment. Although, there are various signs of upward fixation of the patella, but the lameness immediately after the rest is most typical sign [4]. This disease causes subtle extension of the limb, phalangeal flexion so that the animal drags the tip of the hoof (Figure-1). Hereditary, nutritional deficiency, over exploitation, breed, external traumas, intense contraction of the crural triceps muscle and morphological changes in stifle joint are the major potential factors for this condition [2]. It is more common in young and debilitated animals. Diagnosis is based on history, clinical signs and the local palpation of the stifle joint. Medial patellar desmotomy (MPD) is frequently used surgical treatment of dorsal patellar fixation, which can be performed by either open or closed approach [5]. These two different surgical techniques of MPD can be performed in two different restraining positions (in lateral recumbency and in standing position) of animals [6]. The solution of tincture iodii mitis (2.5%) or sodium salicylate has been injected into the joint capsule of stifle joint for treatment of upward fixation of the patella. However, satisfactory results were not obtained in all the cases [7,8]. In present study, the efficacy of the MPD by closed method in lateral recumbency was evaluated on 115 clinical cases. The difference in the incidence among cattle and buffaloes, influence of age, sex and seasons were also studied.

## Materials and Methods

### Ethical approval

Ethical approval was not necessary for clinical cases. All the cases under this research were treated and examined as per standard treatment and examination protocols.

### Animals

This study was conducted on 69 cattle, and 46 buffaloes affected with upward fixation of the patella involving one or both limbs. The disease was clinically diagnosed in animals of different breeds, sexes, age, usage and reproductive statuses.

### Surgery

All the animals were restrained in lateral recumbency with the affected limb towards the ground, and the upper unaffected hind limb was drawn forward and tied with the forelimbs. The limb to be operated was extended, tractioned and held in position by a rope. The tibia-femoral-patellar articulation area was aseptically prepared for surgery. Local analgesia was achieved by infiltrating 5-10 ml of 2% of Lignocaine hydrochloride into the gap located between tibial crest and the medial and intermediate patellar ligaments. On the medial aspect of stifle joint; the site was located by index finger moving upward along the cranial border of the tibia till cranial tibial tuberosity was reached. Middle ligament was traced as it is broadest and thickest among the three patellar ligaments. The finger was slipped inward at the level of medial condyle of tibia into the groove between middle and medial ligaments. The medial ligament was felt as a prominent cord. To employ closed technique of MPD, a small stab incision was made by a no. 11 Bard-Parker (BP) blade and the medial patellar ligament was cut. One or two cross mattress sutures were placed on wounds if it was needed. Post-operatively, enrofloxacin was administered intramuscularly at a dose rate of 5 mg/kg body weight for 5 days. A daily antiseptic dressing was done with dilute liquid povidone iodine solution until wound healing. The placed sutures were removed on 12^th^ postoperative day. The animals were observed for recovery and complications during the study period.

## Results and Discussion

Aforementioned cases were treated by closed approach of MPD. Among 115 cases, 60 were bullocks (52.17%), 9 were cows (7.82%) and 46 were buffaloes (40%). Results of this study were consistent with the findings of earlier workers [9,10]. Contrary to our findings, some workers observed a higher incidence in buffaloes than in cattle [11]. This condition was more prevalent in pregnant, calved and lactating cows and buffaloes aged 4-6.5 years. Buffaloes either in late pregnancy or early lactation are commonly affected by this ailment [8]. Further, this condition was more commonly observed in animals with low body condition score. Both the sexes were equally affected [12]. In the present study, higher incidence was noticed in bullocks, and she buffaloes because these animals, being productive and working animals, receive farmers’ attention earlier. The disease was not seen in male buffaloes as they are slaughtered at an early age for food. Sporadic occurrences are seen in dairy cattle and it is reported to be quite common with draft bullocks in India [13].

The disease was not recorded in crossbred animals, suggesting that pure bred Indian zebu cattle are hereditarily predisposed. Gadgil *et al*. [14] relate the disease to a hereditary factor by tracing the pedigree of Kankrej bullocks. Similarly, chromosomal aberrations were more frequent in affected Egyptian buffaloes when compared to normal buffaloes [15]. Dhillon *et al*. [16] reported that buffaloes in late pregnancy and early lactation suffer from molybdenum induced phosphorus deficiency, and hemoglobinurea were more susceptible to this condition.

Laxity of the patellar ligaments predisposes the animal to upward fixation of the patella. Relaxed ligaments allow the patella to glide freely on the articular surface of the trochela. If the limb is overextended due to muscular cramps or a conformation defect, the patellar apex may get jammed between the trochlear ridges. This would lead to complete extension of the limb in bovine [17].

A greater number of cases were presented in winter 55 (47.82%) followed by rainy season 40 (34.78%) and least in summer 20 (17.39%). The study revealed that the disease exists throughout the year, but the symptoms are exaggerated in winter. Occupational trauma, age of animal or climatic conditions act only as secondary factors to aggravate the signs following development of the condition. Hence, signs are more sever during winter and summer and in draught purpose animals [18]. During the postoperative period, 108 (93.91%) cases recovered uneventfully while complications occurred in 7 (6.08%) cases. Animals, which were put back to work within 1 week of post-surgery showed complications such as lameness, partial dehiscence of the wound and abscesses/pus formation in the wounds [19]. The Buffaloes should be at rest for 2 weeks and prevented from swimming in water to prevent infection of the wound [20]. The technique employed did not require special instruments and could be performed quickly. However, it requires good experience and greater care. Whereas, an open method is time consuming and requires comparatively big surgical incision. It may induce infection to the wound and increases the chances of failure of the operation.

To establish the diagnosis of dorsal patellar fixation, anamnesis, clinical observations and the local palpation were enough without the need for radiography or ultrasonography. During operation, maintaining the animals in lateral recumbency with the affected limbs extended proved to be a safe method, facilitating the execution of the technique and diminishing the risks of accidents during the operation. The only disadvantage was the positional discomfort for the animals and surgeon. The lateral recumbency position, allied to palpation using the thumb, index, and medium fingers helped to localize the medial patellar ligament, which facilitated the surgical technique.

**Figure-2:**
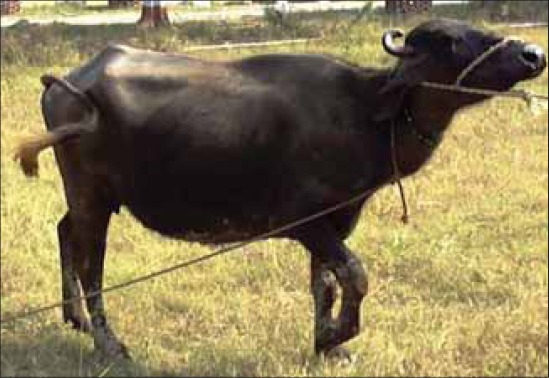
Animal walking normally immediately after surgery.

MPD conducted by the closed method in the lateral recumbency was successful in eliminating the signs of lameness (Figure-2) in 108 (93.91%) out of 115 cases. This showed that the lateral recumbency method of restraining animals can be selected for better results. Mejbah-uddin *et al*. [21] opined that for the MPD, stab (close/blind) method is preferable because there are either little or no haemorrhage with a small hole from exterior, not involve suturing, rapid healing with less post-operative complications no need to give complete rest for a longer period, minimum cost of treatment and finally less time consuming. No complications such as accidental severing of adjacent ligaments were observed. However, Naveen *et al*. [7] reported hematoma and abscess formation in early postoperative period. Baird *et al*. [22] reported success in 25 (89.28%) out of 28 cases after a follow-up. Some workers preferred the open method of patellar desmotomy in the casting position [23]. However in the present study, a high success rate was obtained with the closed method in lateral recumbency. Lateral recumbency was chosen for MPD because it is difficult to control vicious Indian bullocks and perform surgery in the standing position. Even buffaloes were found well suited for lateral recumbency method of operation. The lateral recumbency method showed advantages of accuracy and elimination of the risk of injuries during operation. Contrary to our findings, Shivaprakash and Usturge [24] opined that MPD by the closed method in the standing position is to be preferred than other methods for better results as the ligament appeared more taut and could be accurately cut. Cutting of ligament close to its insertion yielded the best results as the risk of entering the joint cavity was avoided, and this technique avoided the herniation of fat. No groove directors were required for lateral recumbency method in the present study as a curved pointed BP blade itself can be safely inserted behind the ligament. However, techniques of open or closed method or standing or recumbent methods are being practiced depending upon the surgeon’s experience and skill. MPD was successful in complete elimination of the clinical signs of lameness in the affected bovines, when the cases were presented early [25].

## Conclusion

The surgical technique of MPD with the animal restrained in lateral recumbency was effective and can be performed easily. Possible triggering factors and breed predisposition were not observed. The bullocks were more prone to upward fixation of patella and symptoms were exaggerated in winter season. Nutritional deficiency seems to be the most important factor affecting its pathogenesis.

## Authors’ Contributions

All the authors participated in the study and collected data. AKS prepared manuscript. AKG, KSD and HNS revised the manuscript. All authors read and approved the final manuscript.
